# *CYLD*-mutant cylindroma-like basaloid carcinoma of the anus: a genetically and morphologically distinct class of HPV-related anal carcinoma

**DOI:** 10.1038/s41379-020-0584-2

**Published:** 2020-05-27

**Authors:** Erik A. Williams, Meagan Montesion, Radwa Sharaf, James Corines, Parth J. Patel, Brendan J. Gillespie, Dean C. Pavlick, Ethan S. Sokol, Brian M. Alexander, Kevin Jon Williams, Julia A. Elvin, Jeffrey S. Ross, Shakti H. Ramkissoon, Amanda C. Hemmerich, Julie Y. Tse, Mark C. Mochel

**Affiliations:** 1 Foundation Medicine, Inc., 150 Second Street, Cambridge, MA 02141 USA; 2 Department of Pathology, State University of New York Upstate Medical University, 766 Irving Avenue, Syracuse, NY 13210 USA; 3 Department of Surgery, Lewis Katz School of Medicine at Temple University, Philadelphia, PA 19140 USA; 4 Department of Physiology and Medicine, Lewis Katz School of Medicine at Temple University, Philadelphia, PA 19140 USA; 5 Wake Forest Comprehensive Cancer Center and Department of Pathology, Wake Forest School of Medicine, Winston-Salem, NC 27157 USA; 6 Department of Pathology & Laboratory Medicine, Tufts University School of Medicine, 145 Harrison Ave, Boston, MA 02111 USA; 7 Departments of Pathology and Dermatology, Virginia Commonwealth University School of Medicine, Richmond, VA 23298 USA

**Keywords:** Cancer genomics, Tumour virus infections

## Abstract

Rare reports of anal carcinoma (AC) describe histologic resemblance to cutaneous cylindroma, but mutations in the tumor suppressor *CYLD*, the gene responsible for familial and sporadic cylindromas, have not been systematically investigated in AC. Here, we investigate *CYLD*-mutant AC, focusing on molecular correlates of distinct histopathology. Comprehensive genomic profiling (hybrid-capture-based DNA sequencing) was performed on 574 ACs, of which 75 unique cases (13%) harbored a *CYLD* mutation. Clinical data, pathology reports, and histopathology were reviewed for each *CYLD*-mutant case. The spectrum of *CYLD* mutations included truncating (*n* = 50; 67%), homozygous deletion (*n* = 10; 13%), missense (*n* = 16; 21%), and splice-site (*n* = 3; 4%) events. Compared with *CYLD*-wildtype AC (*n* = 499), *CYLD*-mutant ACs were significantly enriched for females (88% vs. 67%, *p* = 0.0001), slightly younger (median age 59 vs. 61 years, *p* = 0.047), and included near-universal detection of high-risk HPV sequences (97% vs. 88%, *p* = 0.014), predominantly HPV16 (96%). The *CYLD*-mutant cohort also showed significantly lower tumor mutational burden (TMB; median 2.6 vs. 5.2 mut/Mb, *p* < 0.00001) and less frequent alterations in PIK3CA (13% vs. 31%, *p* = 0.0015). On histopathologic examination, 73% of *CYLD*-mutant AC (55/75 cases) showed a striking cylindroma-like histomorphology, composed of aggregates of basaloid cells surrounded by thickened basement membranes and containing characteristic hyaline globules, while only 8% of *CYLD*-wildtype tumors (*n* = 34/409) contained cylindroma-like hyaline globules (*p* < 0.0001). *CYLD*-mutant carcinomas with cylindroma-like histomorphology (*n* = 55) showed significantly lower TMB compared with *CYLD*-mutant cases showing basaloid histology without the distinctive hyaline globules (*n* = 14) (median 1.7 vs. 4.4 mut/Mb, *p* = 0.0058). Only five *CYLD*-mutant cases (7%) showed nonbasaloid conventional squamous cell carcinoma histology (median TMB = 5.2 mut/Mb), and a single *CYLD*-mutant case showed transitional cell carcinoma-like histology. Within our cohort of ACs, *CYLD* mutations characterize a surprisingly large subset (13%), with distinct clinical and genomic features and, predominantly, a striking cylindroma-like histopathology, representing a genotype-phenotype correlation which may assist in classification of AC.

## Introduction

Most cancers of the anal and perianal region are squamous cell carcinomas (SCC), predominantly HPV-related [1–4]. Previous publications have reported rare occurrences of anal carcinomas (AC) with “adenoid cystic carcinoma-like” or “cylindroma-like” histologic features, including dense hyaline inclusions of basement membrane material within tumor lobules resembling cutaneous cylindroma [5–7]. The current WHO classification of digestive system tumors acknowledges basaloid patterns in AC, including rare adenoid cystic carcinoma-like histologic features, but describes “the abandonment of histologic subtyping for clinical purposes” owing to intratumoral heterogeneity, limited sampling in small biopsies, and subjectivity [1].

Here, we hypothesized that a molecular approach would allow us to identify these cases and improve the classification system. To address our hypothesis, we chose to focus on mutations in the tumor suppressor *CYLD*, the gene responsible for both familial [8] and sporadic [9] cylindromas. Recent molecular genetic analysis of HPV-positive head and neck SCC has shown the significance of *CYLD* mutation in HPV-driven neoplasia [10]. CYLD normally functions as a deubiquitinating enzyme that negatively regulates the NF-κB [11, 12] and c-Jun N-terminal kinase pathways [13–15]. A study of HPV-positive head and neck SCCs found that *CYLD*-mutant cases showed increased NF-κB activity, episomal HPV DNA instead of integrated HPV DNA, and improved survival, although histopathologic data were not reported [10].

In the current study, we performed a retrospective analysis of AC samples analyzed by comprehensive genomic profiling (CGP) for tumors with *CYLD* mutations. Surprisingly, 13% of our cohort of ACs carried *CYLD* mutations, and those tumors show near-universal detection of high-risk HPV sequences, low tumor mutational burden, and, predominantly, a striking cylindroma-like histopathology with characteristic hyaline globule inclusions.

## Materials and methods

### Cohort and genomic analyses

The Western Institutional Review Board (Protocol No. 20152817) approved this study, including an informed consent waiver and a HIPAA waiver of authorization. A database of cases, which had undergone CGP as part of routine clinical care, performed in a Clinical Laboratory Improvement Amendments-certified, College of American Pathologists-accredited laboratory (Foundation Medicine, Inc., Cambridge, MA, USA), was queried for cases of AC with *CYLD* mutation. The pathologic diagnosis of each case was confirmed on routine hematoxylin and eosin (H&E)-stained slides before DNA extraction. Sections were macrodissected to achieve an estimated percent tumor nuclei (%TN) above 20% in each case, where %TN = 100 times the number of tumor cells divided by total number of nucleated cells. For genomic analysis, ≥60 ng DNA was extracted from 40-μm sections of tumor samples in formalin-fixed, paraffin-embedded tissue blocks. The samples were assayed by adaptor ligation hybrid capture, performed using the Foundation One T7 baitset [16]. All sequenced genes are listed in Supplemental Table 1. Sequencing of captured libraries was performed using the Illumina HiSeq 4000 System to a mean exon coverage depth of targeted regions of >500×, and sequences were analyzed for genomic alterations, including short variant alterations (base substitutions, insertions, and deletions), copy number alterations (focal amplifications and homozygous deletions), and select gene fusions or rearrangements [16–18]. To maximize mutation detection accuracy (sensitivity and specificity) in impure clinical specimens, sequencing was previously optimized and validated to detect base substitutions at a ≥5% mutant allele frequency (MAF), indels with a ≥10% MAF with ≥99% accuracy, and fusions occurring within baited introns/exons with >99% sensitivity [16]. Tumor mutational burden (TMB, mutations/Mb) was calculated based on sequences of 0.8–1.1 Mbp of DNA [18]. Up to 114 loci were assessed for microsatellite instability determinations [19]. For detection of HPV genome sequences, BLASTn comparison of de novo assembly of nonhuman sequencing reads was performed against all viral nucleotide sequences in the comprehensive NCBI RefSeq database. Various HPV types were assessed, including HPV6, 11, 16, 18, 26, 31, 33, 35, 39, 40, 42, 43, 44, 45, 51, 52, 53, 54, 55, 56, 58, 59, 61, 62, 64, 66, 67, 68, 69, 70, 71, 72, 73, 81, 82, 83, 84, CP6108, and IS39. HPV types were classified as described by Muñoz et al. [20], with HPV16, 18, 33, and 58 labeled high risk. HPV(+) designation required contigs ≥80 nucleotides in length with ≥97% sequence identity to the BLAST database sequence.

### Mutational signatures

Characterization of mutational signatures was performed for all specimens with at least 20 nondriver somatic missense alterations. Mutational signatures were designated by trinucleotide context analysis using the Sanger COSMIC cancer mutational signatures [21]. A positive signature required a sample to have at least a 40% fit to a known mutational process, including APOBEC overexpression, exposure to ultraviolet light, hypofunction of the BRCA tumor suppressor, and defects in mismatch repair [21].

### Germline prediction

Identification of *CYLD* mutations as likely to be germline, rather than somatic, was performed using a validated somatic-germline-zygosity algorithm as previously described [17]. In brief, the alignment of sequencing reads and mutant allele frequencies for mutations detected by the Foundation Medicine sequencing pipeline was compared with the expected values produced by the copy number model [16]. Based on this comparison, the algorithm generated a prediction of whether the variant was germline, somatic, or ambiguous [17]. This computational method was utilized instead of the optimal approach of comparative sequencing of paired normal tissue, which was unavailable.

### Clinical-pathological analysis of anal carcinoma cohort harboring *CYLD* mutation

Tumor samples for CGP (Foundation Medicine, Cambridge, MA, USA) had been collected from patients receiving clinical care at other institutions. Clinical-pathological data, including patient age, gender, tumor site, and AJCC stage (8th edition) [22] were collected from accompanying pathology reports.

H&E stained sections from each *CYLD*-mutant case were assessed retrospectively by three pathologists (EAW, JYT, MCM), with the exception of a single case where slides were not available. Histologic features were evaluated, including growth pattern (diffuse, nodular, corded, etc.), glandular differentiation, peripheral palisading of nuclei, presence and arrangement of basement-membrane material, necrosis, presence of squamous differentiation (squamous pearls or eddies), and cytomorphology (basaloid, squamoid, etc.). Accompanying pathology reports were utilized for diagnostically corroborating details, including immunohistochemical findings. For comparison, histologic sections from 409 available *CYLD*-wildtype AC were also evaluated.

Quantitative data were analyzed using the Fisher exact test owing to the categorical quality of the data and the size of the cohort. For comparing age and TMB between two groups, the nonparametric Mann Whitney U test was used. A two‐tailed *P* value of < 0.05 was considered statistically significant, and the Bonferroni correction was applied for multiple simultaneous comparisons (Table 1).

**Table 1:** Comparative demographics, genomic alterations, and additional biomarkers of anal carcinomas stratified by *CYLD* mutation status, with *p* values.

	*CYLD*-mutant	*CYLD*-wildtype	*P*
Number of cases	75	499	
**% female**	**88%**	**67%**	**0.0001**
Median age (range)	61 (41-82)	59 (25-87)	0.047
**Cylindroma-like histology (%)**	**73% (55/75)**	**8% (34/409)**	**<0.0001**
**Sequenced from liver metastasis (%)**	**39% (29/75)**	**18% (91/499)**	**0.0002**
**Sequenced from lung metastasis (%)**	**20% (15/75)**	**7% (35/499)**	**0.0008**
**Median TMB (Q1-Q3; mut/Mb)**	**2.6 (1.7-5.2)**	**5.2 (2.6-7.8)**	**<0.00001**
High-risk HPV positive	97%	88%	0.014
MSI high	0%	1%	1.000
PI3K/AKT/mTOR pathway			
*PIK3CA*	**13%**	**31%**	**0.0015**
*KMT2D*^a^	12%	19%	0.150
*PTEN*	11%	16%	0.302
*FBXW7*	5%	14%	0.041
*STK11*	1%	5%	0.232
*SOX2*^a^	1%	10%	0.008
Epigenetic regulation			
*KMT2C*	7%	15%	0.050
*EP300*	1%	6%	0.159
*KDM6A*	4%	5%	1.000
*BAP1*	1%	3%	0.712
DNA damage			
*TP53*	9%	12%	0.568
Single pass transmembrane receptor			
*FAT1*	3%	9%	0.048
*NOTCH1*	5%	6%	1.000
Cell cycle regulation			
*CCND1* (11q13.3)	1%	7%	0.072
*CDKN2A*	1%	6%	0.106
*TERT* promoter	1%	5%	0.157
*RB1*	7%	4%	0.355
Wnt signaling			
*CTNNB1*	4%	3%	0.452
*APC*	4%	2%	0.410
Receptor tyrosine kinase			
*FGFR3*	3%	4%	0.755
*EGFR*	1%	3%	0.706
RAS/MAPK pathway			
*KRAS*	4%	3%	0.477
*BRAF*	3%	<1%	0.085

The Bonferroni correction for 25 multiple simultaneous comparisons was applied; rows with a significant corrected *p* value threshold (<0.05/25 = 0.002) are in bold.

^a^Limited data in literature on role in PI3K/AKT signaling.

### Review of publicly available dataset for corroboration in an independent cohort

The AACR Project GENIE Consortium dataset (v7.0-public) [23] was interrogated for AC with mutations in *CYLD*.

## Results

### Clinical-pathologic features

Of 574 consecutive nonadenocarcinoma AC specimens, 75 distinct cases (13%) featured *CYLD* mutations. Compared with the rest of the AC cohort, the *CYLD*-mutant group tended to be slightly younger (median 59 vs. 61 years, *p* = 0.047) and was significantly enriched for female gender (88% vs. 67%, *p* = 0.0001) (Table 1). Nearly all cases were clinically advanced: most cases were stage IV (*n* = 63/75; 84%), while the remaining documented cases were stage IIA-B (*n* = 5) and IIIA-B (*n* = 3). Stage was unknown for four cases.

HPV status and typing were determined on all 574 patient samples. *CYLD*-mutant cases showed near-universal detection of high-risk HPV sequences (73 out of 75 cases; 97%), predominantly HPV16 (70/73; 96%). A single HPV16 case had concurrent HPV33 reads, and the remaining three high-risk HPV cases contained HPV18 (*n* = 2) and HPV58 (*n* = 1) reads. *CYLD*-mutant cases more frequently contained high-risk HPV reads compared with *CYLD*-wildtype cases (97% vs. 88% [440/499], *p* = 0.014) (Table 1). *CYLD*-wildtype HPV-positive AC cases (*n* = 440) also predominantly contained HPV16 reads (*n* = 388/440; 88%).

Samples from the 75 *CYLD*-mutant cases consisted of 19 primary AC, two primary-site recurrences, and 54 metastatic disease samples. Of the metastatic samples, the majority were from liver (*n* = 29/54, 54%), followed by lung (*n* = 15), distant lymph nodes (*n* = 3), a regional lymph node (*n* = 1), spleen (*n* = 1), bone (*n* = 1), duodenum (*n* = 1), omentum (*n* = 1), peritoneum (*n* = 1), and sacral soft tissue (*n* = 1). Compared with the *CYLD*-wildtype AC cases in our cohort, *CYLD*-mutant cases were sequenced much more frequently from metastases to the liver (39% [29/75] vs. 18% [91/499], *p* = 0.0002) and lung (20% [15/75] vs. 7% [35/499], *p* = 0.0008). Of the 75 *CYLD*-mutant AC samples, 56 consisted of core biopsies or small incisional biopsies, while 19 were excisional specimens.

Histopathologic examination of the *CYLD*-mutant carcinomas revealed a distinctive and predominant cylindroma-like pattern (55/75 cases; 73%). Other cases showed basaloid morphology with squamous eddies (5/75; 7%) and basaloid morphology without cylindroma-like features or discrete foci of squamous differentiation (9/75; 12%). The remaining cases lacked basaloid cytomorphology and consisted of five conventional SCC with prominent keratinization and one carcinoma with transitional cell carcinoma-like features.

**Fig. 1:**
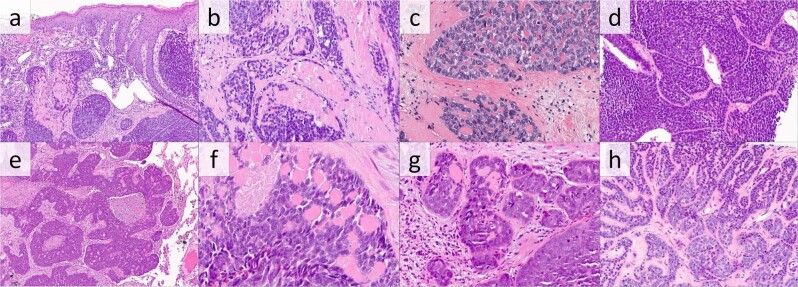
Anal carcinomas with *CYLD* mutation and cylindroma-like histopathologic features. **a** Histopathologic examination reveals a carcinoma involving the dermis composed of rounded lobules of basaloid cells with hyaline inclusions and focally thickened basement membrane (H&E, 100×). **b** Higher power examination of this tumor reveals foci of necrosis and hyaline globules (H&E, 200×). **c** Carcinoma with hyaline globules and associated thickened basement membrane (H&E 400×) **d** Carcinoma with prominent jigsaw-like arrangement of basaloid cells with peripheral palisading, surrounding thickened basement membrane, and small hyaline globules (H&E, 200×). **e, f** This metastatic carcinoma involving the lung is composed of irregular lobules of basaloid cells with central necrosis and focal basement membrane inclusions (H&E, 100×, and 400×). **g** Carcinoma with pleomorphic basaloid cells with focal hyaline globules (H&E, 200×) (**h**) Carcinoma with thickened basement membrane and hyaline globules, also showing biphasic admixture of basaloid cells and cells with paler nuclei (H&E, 200×).

The predominant pattern, cylindroma-like histomorphology, comprised a large majority of *CYLD*-mutant AC (Fig. 1a–h). Histologic slides from 54 cases showed this pattern, while the pathology report from the single case without available slides for our review described matching features. Retrospective histologic examination of the 54 available cases revealed basaloid cells arranged in closely apposed, round to irregular tumor lobules that contained small, round, glassy, and eosinophilic inclusions of surrounding thickened basement membrane, reminiscent of cylindroma. While the basement membrane inclusions were present in all tumors in this subgroup, the inclusions were abundant in 44 cases (81%) (Fig. 1c, h) and focal in the other 10 cases (19%) (Fig. 1f, g). The majority of the *CYLD*-mutant cylindroma-like AC cases showed necrosis (37 cases, 69%), which was usually present in the center of tumor lobules in a comedonecrosis pattern (Fig. 1a, b, e). Only 10 of the *CYLD*-mutant cylindroma-like cases (19%) showed prominent peripheral palisading (Fig. 1d). Cytomorphology was basaloid in all *CYLD*-mutant cylindroma-like cases. Cytology was generally monomorphic, with only five cases (9%) showing biphasic cytology with an admixture of basaloid cells and cells with slightly more cytoplasm and paler nuclei (Fig. 1h). Cellular pleomorphism was assessed to be mild in 40 cases (74%), moderate in 12 cases (22%), and severe in two cases (4%). Rare squamous eddies were seen in three cases; otherwise, none demonstrated the presence of squamous eddies or pearls.

Because the cylindroma-like histomorphology within the *CYLD*-mutant group was striking and potentially distinctive, we reviewed the histopathology from all 409 available *CYLD*-wildtype AC in our database for comparison. Of the *CYLD*-wildtype cases, 407 were SCC, including histologic variants, and two were adenocarcinomas. Only 8% (*n* = 34) of the *CYLD*-wildtype cases showed aggregates of tumor cells containing round fragments of hyalinized material, consistent with basement membrane inclusions, imparting a cylindroma-like appearance similar to the *CYLD*-mutant cases (Supplemental Fig. 1). These inclusions were abundant in 11 cases (32%) and focal in 23 cases (68%). The cytomorphology of *CYLD*-wildtype, cylindroma-like cases were predominantly basaloid (29 cases, 85%), although five cases (15%) consisted largely of squamoid cells with more voluminous, densely eosinophilic cytoplasm (Supplemental Fig. 1d). Importantly, compared with *CYLD*-wildtype AC, *CYLD*-mutant AC showed over ninefold higher frequency of cylindroma-like histology, as principally designated by the presence of basement membrane inclusions within tumor cell aggregates (73% vs. 8%, *p* < 0.0001; Table 1).

**Fig. 2:**
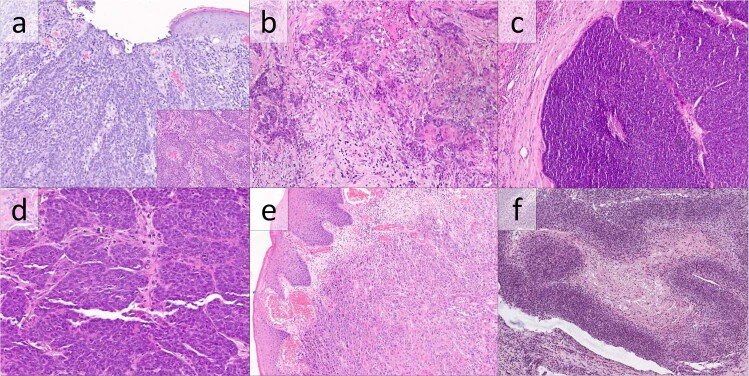
Anal carcinomas with *CYLD* mutation lacking cylindroma-like basement membrane inclusions. (**a and inset**) Anal carcinoma composed predominantly of irregular aggregates of basaloid cells showing rare continuity with surface epithelium (**a**) and focal squamous eddies (**inset**) (H&E, 200×). **b** Metastatic anal basaloid carcinoma involving a lymph node with infiltrative small aggregates of basaloid cells with occasional squamous morules (H&E 200×). **c** Basaloid anal carcinoma, metastatic to liver, consisting of large aggregates of basaloid tumor cells without apparent basement membrane material or squamous differentiation (H&E, 100×). **d** Basaloid anal carcinoma with small, closely apposed aggregates of basaloid tumor cells without apparent basement membrane inclusions or squamous differentiation (H&E, 200×). **e** Anal carcinoma composed of aggregates of cells with glassy eosinophilic cytoplasm, consistent with conventional squamous cell carcinoma (H&E, 100×). **f** A single case showed thickened trabeculae of basaloid carcinoma cells with a transitional cell carcinoma-like appearance (H&E, 100×).

Five *CYLD*-mutant AC cases (7%) were composed of aggregates of basaloid cells with squamous eddies (Fig. 2a, b). Squamous eddies, defined as rounded whorls of squamous cells with eosinophilic cytoplasm, were extensive in one case, focal in three cases, and rare in one case. A jigsaw-like arrangement of tumor lobules was present in two cases, while peripheral palisading was prominent in two. Necrosis was extensive in two cases and focal in one. No cases contained basement membrane inclusions or prominent basement membranes. Histologic features of squamous differentiation were limited to squamous eddies: squamous pearls were not identified, and intercellular spines were inconspicuous. Cellular pleomorphism was mild in all cases.

Nine *CYLD*-mutant cases (12%) consisted of basaloid cells with neither basement membrane inclusions nor squamous differentiation (Fig. 2c, d). Of these, six showed a jigsaw-like arrangement of tumor lobules, two showed prominent peripheral palisading, and four showed necrosis. Cellular pleomorphism was mild in three cases and moderate in six.

The remaining six cases of *CYLD*-mutant AC all lacked basaloid cytomorphology. Five cases (7%) showed histologic features of conventional squamous cell carcinoma (Fig. 2e), with nodular proliferations of cells with dense, pink cytoplasm and severe nuclear pleomorphism. The remaining case showed transitional cell carcinoma-like histology, a recognized histologic pattern in AC [7], with thick trabeculae of tumor cells and abundant necrosis (Fig. 2f).

Glandular differentiation was not seen in any of the *CYLD*-mutant AC cases. The mucinous microcytic pattern, previously reported in AC [7] was not observed, and clear cell changes were not present in any of the *CYLD*-mutant cases.

Although immunohistochemical slides were not available for confirmatory review by the authors, accompanying pathology reports stated that the *CYLD*-mutant ACs that were evaluated by immunohistochemistry were nearly always positive for p63 (30/31 cases), p40 (15/16 cases), CK5/6 (26/27 cases), and p16 (24/24 cases). In contrast, CK7 was diffusely positive in only 6/20 cases, focally positive in 4/20 cases, and negative in the remaining 10 cases. *CYLD*-mutant ACs were uniformly negative when stained for synaptophysin (*n* = 24 cases), chromogranin (*n* = 20), CK20 (*n* = 20), CDX2 (*n* = 13), and TTF1 (*n* = 13).

### Comprehensive genomic profiling

**Fig. 3:**
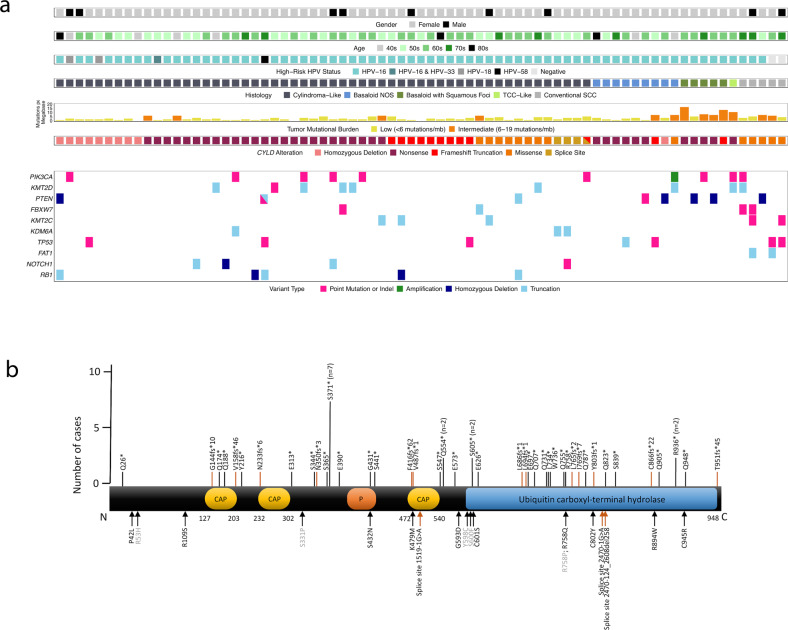
Clinicopathologic features and molecular landscape of *CYLD*-mutant anal carcinoma. **a** Tile plot to summarize clinical features, histopathology, and molecular alterations in *CYLD*-mutant anal carcinoma. **b** Schematic of functional domains of *CYLD* (transcript NM_015247), to include identified mutation sites (79 mutations in 75 cases). The resulting terminal amino acid location for nonsense (*n* = 38) and frameshift mutations (*n* = 12) in *CYLD* are denoted by black and orange bars, respectively (top of diagram). Missense mutations (*n* = 16) and splice site mutations (*n* = 3), each identified in single case, are labeled with black and orange arrows, respectively (lower diagram). Mutations in gray font were identified in cases with conventional squamous cell carcinoma histology. Ten cases with homozygous deletions in *CYLD* are not shown in (**b**). CAP cytoskeleton-associated proteins, P phosphorylation region.

*CYLD* mutations included truncating (*n* = 50), homozygous deletion (*n* = 10), missense (*n* = 16), and splice site (*n* = 3) mutations (Fig. 3a, b). Truncating mutations occurred in exons 4 (*n* = 2), 5 (*n* = 5), 8 (*n* = 1), 9 (*n* = 10), 10 (*n* = 5), 11 (*n* = 3), 12 (*n* = 3), 13 (*n* = 1), 15 (*n* = 3), 16 (*n* = 4), 17 (*n* = 3), 18 (*n* = 3), 19 (*n* = 2), and 20 (*n* = 5). Missense mutations occurred in exons 4 (*n* = 3), 8 (*n* = 1), 10 (*n* = 2), 12 (*n* = 4), 15 (*n* = 1), 17 (*n* = 2), 18 (*n* = 1), 19 (*n* = 1), and 20 (*n* = 1).

Figure 3a and Table 1 show the most frequent genomic alterations outside the *CYLD* locus in the *CYLD*-mutant vs. *CYLD*-wildtype AC cohort. The *CYLD*-mutant group showed significantly lower TMB and less frequent alterations in *PIK3CA* (Table 1).

Cases with cylindroma-like histomorphology (*n* = 55) showed significantly lower TMB compared with cases with basaloid histology lacking cylindroma-like features (*n* = 14) (1.7 vs. 4.4, *p* = 0.0058). In addition, cases with basaloid histology lacking cylindroma-like features showed a higher frequency of *PTEN* alterations (29% [4/14] vs. 5% [3/55], *p* = 0.0273). Demographics of cylindroma-like and noncylindroma-like basaloid cases were similar.

Further analysis revealed that, compared with cases with basaloid histomorphology without further differentiation (*n* = 9), cases with basaloid appearance with focal squamous differentiation (*n* = 5) had a significantly higher TMB (7.8 vs. 3.5, *p* = 0.0105). No other significant differences were identified.

Compared with the remainder of the *CYLD*-mutant cohort, cases with conventional squamous cell carcinoma histology with prominent keratinization (*n* = 5 cases) were associated with HPV-negative status (40% [2/5] vs. 0% [0/70], *p* = 0.0036) and showed elevated TMB (median = 5.2 vs. 2.6, *p* = 0.0242). Cases also showed a significantly elevated frequency of alterations in *FBXW7* (40% [2/5] vs. 2/70 [3%], *p* = 0.0204), *FAT1* (40% [2/5] vs. 0% [0/70], *p* = 0.0036), and *KMT2C* (40% [2/5] vs. 4% [3/70], *p* = 0.0331).

Tumors sequenced from primary (*n* = 21) vs. metastatic sites (*n* = 54) showed similar demographics and percentages of genomic alterations. Median TMB, while slightly lower for tumors sequenced from primary sites, was not significantly different (1.7 vs. 2.6 mut/Mb, *p* = 0.428).

Mutational signatures were obtained for nine *CYLD*-mutant cases. Three cases were identified with an APOBEC signature (two with basaloid histology, one with TCC-like histology). Available *CYLD*-wildtype cases (*n* = 162) showed similar frequency of APOBEC signature (*n* = 91/162).

Comparison of cases with *CYLD* mutations within the ubiquitin-specific protease domain (exons 12–20) and outside the ubiquitin hydrolase domain (exons 4–11) revealed no significant differences.

In total, 17 of the 75 cases had available SGZ algorithm data to predict germline status of *CYLD* alterations. Of the 17 cases, two cases had two *CYLD* mutations each. In total, 18 of the 19 *CYLD* mutations were predicted to be somatic. One case with a single *CYLD* mutation (P42L; allele frequency = 60%) was predicted germline; the case showed cylindroma-like histology.

Comparison of *CYLD*-wildtype AC with vs. without cylindroma-like histology revealed that cylindroma-like cases showed significantly lower frequency of *PIK3CA* (9% [3/34] v. 32% [120/375], *p* = 0.0032) and *PTEN* genomic alterations (3% [1/34] vs. 18% [66/375], *p* = 0.027), and higher frequency of sequencing from samples of liver metastasis (32% [11/34] vs. 17% [63/375], *p* = 0.034). 32 of 34 *CYLD*-wildtype cylindroma-like AC (94%) were positive for high-risk HPV. Thus, in these key parameters, *CYLD*-wildtype cylindroma-like AC resembled *CYLD*-mutant cylindroma-like AC. No other significant differences between *CYLD*-wildtype AC with vs. without cylindroma-like histology were identified.

### Publicly available dataset for corroboration in an independent cohort

The unexpectedly high frequency of *CYLD*-mutant cases in our AC cohort (13%) prompted us to interrogate the AACR Project GENIE Consortium dataset (v7.0-public) [23]. A total of 10 *CYLD*-mutant AC cases were identified (10/83 [12.0%]), nearly identical to our 13%. Median age was 58 years (range 51–80), and the cases were enriched for female gender (*n* = 8/10), as in our cohort. *CYLD* mutations included truncating (*n* = 6), homozygous deletion (*n* = 2), and missense (*n* = 2) mutations. Truncating mutations occurred in exons 9 (*n* = 1), 11 (*n* = 1), 12 (*n* = 1), 17 (*n* = 2), and 18 (*n* = 1). Missense mutations occurred in exons 5 (*n* = 1) and 20 (*n* = 1). As in our cohort, concurrent alterations were each of low frequency: in *PTEN* (*n* = 1 case), *KMT2D* (*n* = 1 case), *BRAF* (*n* = 1 case), *APC* (*n* = 1 case), and *RB1* (*n* = 1 case). All cases were wildtype for *PIK3CA* alterations. Histopathology was not available for review.

## Discussion

Our series connects a distinctive histopathology of cylindroma-like basaloid carcinoma of the anus with mutations in *CYLD*, a gene responsible for both syndromic [8] and sporadic [9] cylindromas, confirming both a morphologic and genetic relationship with cutaneous cylindroma. Furthermore, the close correlation of HPV-positivity and *CYLD* mutation in this context supports the concept that *CYLD* mutation contributes to AC primarily through HPV-related carcinogenesis.

Previous studies have reported rare anal basaloid carcinomas with “adenoid cystic carcinoma-like” histologic features, including dense globules of basement membrane material within tumor lobules [5–7]. Among these histopathologic studies, Chetty et al. [6] described two cases that closely resemble the cases in our series. Those authors noted both the “jigsaw” pattern of intimately admixed tumor lobules and the presence of basement membrane material highly reminiscent of cylindroma. Photomicrographs from their article [6] and a later article by Graham et al. [7] show prominent eosinophilic, glassy thickening of the basement membrane surrounding tumor lobules with small round inclusions of the same material within tumor lobules, closely resembling cylindroma-like carcinomas in this study. Interestingly, a detailed case report of spiradenocylindroma-like basaloid carcinoma of the anus showed highly similar histologic features to our cases and those of references [6, 7], as well as HPV positivity, but examination of the *CYLD* gene detected no mutation in the tumor [24]; we similarly identified cylindroma-like carcinomas infrequently among *CYLD*-wildtype ACs. Another recent report described a similar case, designated as cylindroma-like basaloid AC, although molecular studies were not described [25]. Immunohistochemistry results extracted from pathology reports in our cohort were similar to the cases in previous studies [6, 7], with CK5/6, p63, and p16 positivity, and variable CK7 positivity.

Although several investigators have noted the histologic resemblance of reportedly rare ACs to cylindroma [6, 24, 25], to our knowledge, *CYLD* mutation has not been described in detail for AC. Comprehensive molecular genetic studies of AC have shown various mutations in HPV-positive cases, especially those involving the phosphoinositol-3-kinase pathway (including *PIK3CA*, *PTEN*, and *AKT1/2*), while HPV-negative carcinomas often show *TP53* mutation and loss of *CDKN2A* [26–31]. A search of AC in the AACR Project GENIE Consortium dataset [23] found that 12% of ACs harbor *CYLD* mutations, similar to the 13% incidence in our cohort.

CYLD functions as a deubiquitinating enzyme which negatively regulates activity of the NF-κB pathway [11, 12] and c-Jun N-terminal kinase pathway [13–15]. In mouse models, inactivation of CYLD in the epidermis promotes sebaceous hyperplasia and proliferations with basaloid and sebaceous components [32], while global knockout mice are prone to developing cutaneous papillomas [12]. Loss of CYLD activity has been correlated with more aggressive behavior in cutaneous squamous cell carcinoma [33], melanoma [13, 34], pancreatic carcinoma [35], and hepatocellular carcinoma [15]. In addition, gene expression profiling of *CYLD*-mutant tumors has shown dysregulated tropomyosin kinase signaling, which has been suggested as a potential target for therapy [36, 37].

The preponderance of clinical studies of *CYLD* mutation have concerned syndromic manifestations of familial cylindromatosis, Brooke-Spiegler syndrome, and multiple familial trichoepitheliomas. After the initial characterization of *CYLD* mutation in familial cylindromatosis [8], subsequent publications identified a variety of mutations in these cutaneous tumor syndromes [38, 39]. A recent review of the literature described 95 unique syndrome-associated mutations of *CYLD*, most occurring within exons 9–20 [40] and most resulting in protein truncation [40]. *CYLD* mutations are also present in sporadic cylindromas and some spiradenomas [9]. Most *CYLD* mutations in our AC cohort are similar, i.e., occurring within exons 9–20 and resulting in protein truncation. In addition, germline prediction results suggest that the *CYLD* mutations in AC were predominantly somatic.

A recent molecular genetic study of cutaneous cylindromas, spiradenomas, and spiradenocarcinomas found *CYLD* mutations in nearly all cylindromas and some spiradenomas [9]. Most spiradenomas, however, harbored *ALPK1* mutations, which were mutually exclusive from *CYLD* mutations. Spiradenocarcinomas showed frequent oncogenic comutations involving genes such as *TP53*. That study did not investigate cylindrocarcinoma, an extremely rare tumor.

Previously reported cylindrocarcinomas of the skin are not clearly analogous to the cases described in our current series. Cutaneous cylindrocarcinomas of the skin show heterogeneous malignant components, ranging from low grade adenocarcinoma resembling that arising from the salivary gland to sarcomatoid malignant tumors, only identifiable as related to the adnexal tumor by the presence of an intact precursor cylindroma by histologic examination [41]. Prior studies of the genetics of cylindrocarcinoma are limited; investigations have to-date described *TP53* mutations in small numbers of cylindrocarcinomas within larger series of adnexal carcinomas [42, 43].

While *CYLD*-mutated ACs share some histologic features with cutaneous cylindroma, multiple histologic differences are readily apparent. Both tend to show a jigsaw-like arrangement of basaloid cell aggregates with thickened basement membranes and inclusions of basement membrane material. *CYLD*-mutant basaloid carcinomas, however, show permeative growth and frequently display necrosis, often in a comedo pattern, features unusual for cutaneous cylindroma. In addition, while cutaneous cylindromas are typically composed of a dimorphic cell population, with dark cells containing scant cytoplasm at the periphery of tumor lobules and light cells with pale cytoplasm and nuclei centrally, the *CYLD*-mutated basaloid carcinomas in our series were typically composed of monomorphic populations of basaloid cells. Finally, the degree of nuclear irregularity, hyperchromasia, and pleomorphism seen in the *CYLD*-mutant ACs exceeds that of cutaneous cylindroma.

Recent molecular genetic study of HPV-positive head and neck SCC found that one third of cases harbored mutation of either *CYLD* or *TRAF3*, the latter of which also encodes a deubiquitinating enzyme and negative regulator of NF-κB [10]. Among HPV-positive head and neck SCC, *CYLD*- or *TRAF3*-mutant tumors showed increased NF-κB activity, episomal HPV DNA rather than integrated HPV DNA, increased expression of genes related to adhesion, motility, proliferation, and differentiation, and improved survival. *CYLD* and *TRAF3* mutations were found only rarely among HPV-negative head and neck SCC, suggesting that *CYLD* mutation contributes to carcinogenesis mostly through HPV-related pathways. While these studies lacked histomorphologic data which would enable closer correlation with the cases in our cohort, the demonstrated relationship between *CYLD* mutation and HPV-driven carcinogenesis may be analogous to our series of *CYLD*-mutant cylindroma-like ACs.

In this study, the presence of cylindroma-like histologic appearance, as principally defined by the presence of basement membrane inclusions, was significantly correlated with *CYLD* mutation in the context of AC. Cylindroma-like histology had a sensitivity and specificity of 73% and 92%, respectively, for the presence of *CYLD* mutation. Notably, among cylindroma-like cases, hyaline globule inclusions tended to be abundant in the *CYLD*-mutant group and focal in the *CYLD*-wild type group. There were also some cases of *CYLD*-wild type AC with cylindroma-like inclusions, but with more squamoid cytology (Supplemental Fig. 1d), differing from the *CYLD*-mutant cases. The *CYLD*-mutant cases without accompanying cylindroma-like features may be partly attributable to sampling error; most biopsies were partial and may have missed cylindroma-like foci. With respect to the *CYLD*-wildtype cases with cylindroma-like hyaline globule inclusions, we have considered that these cases may contain non-*CYLD*, as-yet uncharacterized mutations activating related pathways to promote a similar histogenesis, although this requires additional study.

The rarity of HPV-negative ACs with *CYLD* mutation suggests that HPV infection is an obligate precursor for *CYLD*-mutant basaloid carcinomas of the anus. Intriguingly, in contrast to cutaneous sites, where cylindromas are far more common than their malignant counterpart, a benign counterpart to cylindroma-like basaloid carcinoma of the anus is not known. To our knowledge, cylindromas, which occur most commonly on the scalp, are extremely rare in the anal or perianal region [44].

*CYLD* mutations also characterize a subset of salivary gland neoplasms: both basal cell salivary adenomas and adenocarcinomas can possess *CYLD* mutations [45]. Interestingly, the basal cell adenomas and carcinomas harbor mutations of *CYLD* in exons 9–11, while only basal cell adenocarcinomas harbor *CYLD* mutations involving exons 12–20, corresponding to the ubiquitin-specific hydrolase domain. Comparison of ACs with *CYLD* mutations in exons 9–11 and exons 12–20 revealed no significant differences. Of note, the rare reported cases of basal cell adenocarcinoma with HPV testing were HPV(−) and, therefore, perhaps not analogous to *CYLD*-mutant AC [46].

Other recent work has refined the understanding of a particular subtype of sinonasal carcinoma, which shows both adenoid cystic carcinoma-like histologic features and HPV-positivity [47, 48]. This carcinoma, designated “HPV-positive multiphenotypic sinonasal carcinoma”, pairs histologic features of a spectrum of salivary gland carcinomas with involvement of surface epithelium [48]. Interestingly, in addition to displaying cribriform patterns highly reminiscent of adenoid cystic carcinoma, some cases also contain foci of hyalinized basement membrane-like material within tumor cell aggregates. *CYLD* mutation status of these lesions has not been described to the authors’ knowledge.

The distinctive nature of our underlying sample set is a limitation of this study. All cases were sent for CGP for detection of targetable genetic alterations. As such, the cases tended towards advanced disease in which surgical therapy alone was not curative. Although we lacked detailed follow-up to definitively characterize the full clinical significance of *CYLD* mutation, we documented a significant preponderance of liver involvement amongst *CYLD*-mutant AC.

The majority of our cases were core biopsies, and, thus, limited sampling may have precluded identification of some histologic features (e.g., additional material from a case with the basaloid pattern without further differentiation may have revealed cylindroma-like features). Although some primary tumor samples contained portions of intact mucosa enabling corroboration of anal site, the anatomical designation of primary site was based principally on accompanying requisition forms and pathology reports. While we reviewed data on immunohistochemistry from accompanying pathology reports, we were not able to review immunohistochemical slides to confirm reported findings or to perform additional immunostains. In addition, while comparative sequencing of normal tissue for definitive exclusion of germline mutation was not available, the SGZ algorithm findings were consistent with somatic mutations in 94% of cases with available data.

*CYLD*-mutant cylindroma-like basaloid carcinomas of the anus represent a significant subgroup of ACs with distinctive genetic and histologic features and near-universal detection of HPV-positivity. These characteristic genetic findings, paired with the striking histopathology, may aid in the subtyping of AC [1]. Our findings represent a genotype-phenotype correlation with potentially important implications for classification of AC. Additional studies are needed to define prognostic features and potential therapeutic approaches [36, 37] to this unique, common, and previously uncharacterized, anal cancer subtype.

## Supplementary information

Supplemental Figure 1

Supplemental Table 1
