# Supplementary material for: CYLD-mutant cylindroma-like basaloid carcinoma of the anus: a genetically and morphologically distinct class of HPV-related anal carcinoma
Source: Mod Pathol. 2020 May 27;33(12):2614–25. doi: 10.1038/s41379-020-0584-2 (PMC7685972; doi:10.1038/s41379-020-0584-2)
Supplement: Supplementary file 1 — Supplemental Figure 1 [file 41379_2020_584_MOESM1_ESM.pdf]

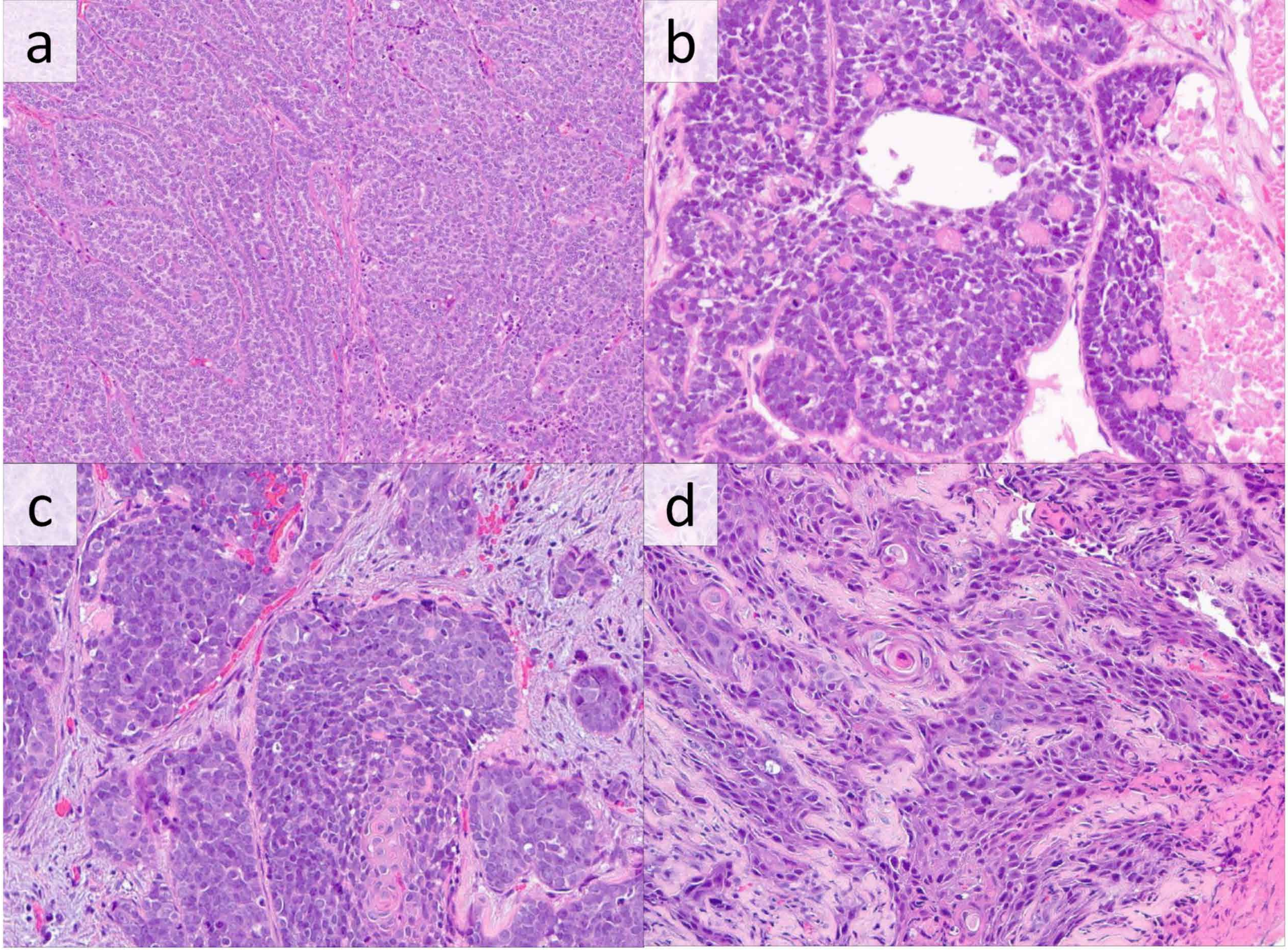

Supplemental Figure 1. *CYLD*-wild type anal carcinomas with cylindroma-like inclusions of hyaline material (**a,b**) Anal carcinoma with closely apposed aggregates of basaloid cells (**a**) with inclusions of round basement membrane material (**b**) (H&E, 100x and 200x). (**c**) Anal carcinoma composed of basaloid cells with focal basement membrane inclusions (H&E 200x). (**d**) Anal carcinoma with irregular aggregates of squamous cells with entrapped basement membrane material (H&E, 200x).
